# Postoperative outcomes in academic *versus* non-academic hospitals: population-based cohort study

**DOI:** 10.1093/bjsopen/zraf090

**Published:** 2025-12-01

**Authors:** Carlos Riveros, Sanjana Ranganathan, Michael Geng, Renil S Titus, Natalie Coburn, Bheeshma Ravi, Yusuke Tsugawa, Vatsala Mundra, Zachary Melchiode, Eusebio Luna Velasquez, Angela Jerath, Allan S Detsky, Christopher J D Wallis, Raj Satkunasivam

**Affiliations:** Department of Urology, Houston Methodist Hospital, Houston, Texas, USA; Department of Urology, Houston Methodist Hospital, Houston, Texas, USA; School of Engineering Medicine, Texas A&M University, Houston, Texas, USA; Department of Urology, Houston Methodist Hospital, Houston, Texas, USA; School of Engineering Medicine, Texas A&M University, Houston, Texas, USA; Department of Urology, Houston Methodist Hospital, Houston, Texas, USA; Division of Surgical Oncology, Department of Surgery, Sunnybrook Health Sciences Center, Toronto, Ontario, Canada; Division of Orthopedic Surgery, Department of Surgery, Sunnybrook Health Sciences Center, Toronto, Ontario, Canada; Division of General Internal Medicine and Health Services Research, David Geffen School of Medicine at UCLA, Los Angeles, California, USA; Department of Health Policy and Management, UCLA Fielding School of Public Health, Los Angeles, California, USA; Department of Urology, Houston Methodist Hospital, Houston, Texas, USA; Department of Urology, Houston Methodist Hospital, Houston, Texas, USA; Department of Urology, Houston Methodist Hospital, Houston, Texas, USA; Department of Anesthesia, Sunnybrook Health Sciences Center, Toronto, Ontario, Canada; Department of Medicine, Mount Sinai Hospital and University Health Network, Toronto, Ontario, Canada; Institute for Health Policy, Management and Evaluation, University of Toronto, Toronto, Ontario, Canada; Department of Medicine, University of Toronto, Toronto, Ontario, Canada; Division of Urology and Surgical Oncology, Department of Surgery, Princess Margaret Cancer Center, University Health Network, University of Toronto, Toronto, Ontario, Canada; Division of Urology, University of Toronto, Toronto, Ontario, Canada; Division of Urology, Mount Sinai Hospital, Toronto, Ontario, Canada; Department of Urology, Houston Methodist Hospital, Houston, Texas, USA

Academic hospitals are consistently ranked higher than non-academic facilities when survival rates and complication rates are used to measure performance^1^. However, the literature examining the association of academic status with postoperative outcomes to support claims of improved quality is conflicting^2,3^. Quality assessment is complicated by factors, some of which improve outcomes (high-volume academic surgeons) whereas others may increase the risk of complications (high case complexity, trainee participation). Analysis of U.S. Medicare data found lower rates of 30-day mortality among patients hospitalized in academic *versus* non-academic hospitals^2^. No studies have explored this over a broad range of surgeries and patients. Thus, a population-level retrospective study was conducted to measure the association between a hospital’s academic status and 30-day, 90-day, and 1-year postoperative outcomes among a broad range of procedures and patients.

In all, 1 165 711 adult patients covered by the Ontario Health Insurance Plan and who underwent 1 of 26 common surgical procedures (*Tables S1*, *S2*; *Fig. S1*) between 2007 and 2021 were analysed. Academic hospitals were identified by Health Force Ontario list^4^ (*Table S3*), which designated academic status by association with a university’s faculty of medicine. The primary outcome was a composite of 30-day postoperative deaths, complications, and readmissions. Secondary outcomes were the composite outcome at 90 days and 1 year, along with individual components of the composite outcome, and length of hospital stay and operative time (*Table S4*). The association between hospital status and outcomes was assessed using multivariable generalized estimating equations accounting for patient, surgeon, anaesthetist, and hospital-level covariates, with clustering on procedure (*Table S5*). An odds ratio > 1 indicated poorer outcomes for patients treated in academic hospitals.

Surgeons at academic hospitals were more likely to be in the highest quartile for annual case volume than surgeons at non-academic hospitals. More cancer and high-complexity surgeries were performed at academic than non-academic hospitals (*Table S1*). After adjusting for these factors, no significant association was founded between academic designation and the odds of the 30-day outcome (adjusted odds ratio (aOR) 1.13; 95% confidence interval (c.i.) 0.99 to 1.28; *Table 1*). Academic designation was associated with an increased risk of 30-day readmission (aOR 1.19; 95% c.i. 1.10 to 1.29), a longer 30-day hospital stay (adjusted relative risk (aRR) 1.19; 95% c.i. 1.10 to 1.28), and longer operative time (aRR 1.31; 95% c.i. 1.19 to 1.44), but not mortality (aRR 1.05; 95% c.i. 0.88 to 1.26; *Table 1*).

**Table 1:** Multivariable generalized estimating equation regression models, with clustering based on procedure fee code for outcomes within 30 days, 90 days, and 1 year of the index surgery for academic *versus* non-academic hospitals

Model no.	Outcome	Outcome within 30 days		Outcome within 90 days		Outcome within 1 year	
		aOR/aRR*	*P*	aOR/aRR*	*P*	aOR/aRR*	*P*
1	Composite endpoint	1.13 (0.99, 1.28)	0.072	1.13 (1.01, 1.27)	0.036	1.14 (1.05, 1.24)	0.003
2	Death	1.05 (0.88, 1.26)	0.579	1.13 (0.95, 1.36)	0.167	1.21 (1.03, 1.43)	0.020
3	Readmission	1.19 (1.10, 1.29)	<0.001	1.18 (1.10, 1.27)	<0.001	1.16 (1.08, 1.25)	<0.001
4	Complications	1.09 (0.91, 1.31)	0.331	1.10 (0.92, 1.31)	0.299	1.10 (0.94, 1.28)	0.224
5	Length of hospital stay	1.19 (1.10, 1.28)	<0.001	1.24 (1.14, 1.36)	<0.001	1.26 (1.15, 1.38)	<0.001
6	Duration of surgery	1.31 (1.19, 1.44)	<0.001	NA	NA	NA	NA

*Data show aOR (for binary outcomes) and aRR (for continuous outcomes) for academic *versus* non-academic hospitals. Values in parentheses are 95% confidence intervals. Generalized estimating equations modelling was used, dealing with clustering based on procedure fee code (logistic regression with binomial distribution with logit link for binary outcomes; negative binomial distribution with log link for continuous outcomes), adjusted for: surgeon age (continuous), sex, annual case volume (quartiles), specialty, and years of practice (continuous); anaesthetist age (continuous), sex, annual case volume (quartiles), and years of practice (continuous); patient age (continuous), sex, and co-morbidity (categorical); rurality (rural *versus* urban); income quintile (quintiles); local health integration network; hospital status (academic *versus* non-academic); and index year. aOR, adjusted odds ratio; aRR, adjusted relative risk; NA, not applicable.

Academic designation was associated with higher odds of the composite 90-day outcome (aOR 1.13; 95% c.i. 1.01 to 1.27) and 1-year outcome (aOR 1.14; 95% c.i. 1.05 to 1.24), driven by readmissions (aOR 1.18 (95% c.i. 1.10 to 1.27) and 1.16 (95% c.i. 1.08 to 1.25) for 90 days and 1 year, respectively). Although the odds of complications did not differ significantly at either time point, patients treated at academic facilities had higher mortality at 1 year (aOR 1.21; 95% c.i. 1.03 to 1.43), but not 90 days. Academic designation was associated with a longer cumulative hospital stay at 90 days (aRR 1.24; 95% c.i. 1.14 to 1.36) and at 1 year (aRR 1.26; 95% c.i. 1.15 to 1.38; *Table 1*). Subgroup and sensitivity analyses demonstrated that the higher likelihood of adverse postoperative outcomes at academic facilities may be further influenced by surgeon age/experience and surgical indication (for example, cancer surgery; *Fig. S2*). When the duration of surgery was added as a covariate, all associations became non-significant (*Tables S7–10*).

In this population-based, multidisciplinary cohort, surgery at academic hospitals was not associated with either decreased or increased statistically significant odds of the composite 30-day outcome. It was associated with significantly increased odds of the composite outcome at 90 days and 1 year, driven by increased odds of readmissions at 90 days and readmission and mortality at 1 year. The difference in 1-year mortality could be due to confounding by increased case complexity or morbidity burden in academic hospitals. There was no difference in the odds for complications at any time point. Similar findings were reported previously using U.S. Medicare data^5^. Although there maybe residual confounding not captured by the model used in this study, the conclusion is that the widely held view that teaching hospitals provide higher-quality surgical care, is not always supported.

## Supplementary Material

zraf090_Supplementary_Data

## Data Availability

Additional/raw data are available upon request from the corresponding author.
