# Supplementary material for: Postoperative outcomes in academic versus non-academic hospitals: population-based cohort study
Source: BJS Open. 2025 Dec 1;9(6):zraf090. doi: 10.1093/bjsopen/zraf090 (PMC12667259; doi:10.1093/bjsopen/zraf090)
Supplement: zraf090_Supplementary_Data [file zraf090_supplementary_data.docx]

**Title:**

**Postoperative Outcomes in Academic Versus Non-Academic Hospitals: Population-Based Cohort Study**

**Authors**

Carlos Riveros, MD^1^; Sanjana Ranganathan, MBE^1,2^; Michael Geng, BS^1,2^; Renil S. Titus, MD^1^; Natalie Coburn, MD, MPH^3^; Bheeshma Ravi, MD, PhD^4^, Yusuke Tsugawa MD, PhD^5,6^; Vatsala Mundra, BS^1^, Zachary Melchiode, MD^1^; Eusebio Luna Velasquez MD^1^, Angela Jerath, MD, MSc^7^; Allan S. Detsky MD, PhD^8,9,10^, Christopher J.D. Wallis, MD, PhD^11,12,13^; Raj Satkunasivam, MD, MS^1^

**Affiliations**

^1^ Department of Urology, Houston Methodist Hospital, Houston, Texas, USA

^2^ School of Engineering Medicine, Texas A&M University, Houston, Texas, USA

^3^ Division of Surgical Oncology, Department of Surgery, Sunnybrook Health Sciences Center, Toronto, Ontario, Canada

^4^ Division of Orthopedic Surgery, Department of Surgery, Sunnybrook Health Sciences Center, Toronto, Ontario, Canada

^5^ Division of General Internal Medicine and Health Services Research, David Geffen School of Medicine at UCLA, Los Angeles, California, USA

^6^ Department of Health Policy and Management, UCLA Fielding School of Public Health, Los Angeles, California, USA

^7^ Department of Anesthesia, Sunnybrook Health Sciences Center, Toronto, Ontario, Canada

^8^ Department of Medicine, Mount Sinai Hospital and University Health Network, Toronto, Ontario, Canada

^9^ Institute for Health Policy, Management and Evaluation, University of Toronto, Toronto, Ontario, Canada

^10^ Department of Medicine, University of Toronto, Toronto, Ontario, Canada

^11^ Division of Urology and Surgical Oncology, Department of Surgery, Princess Margaret Cancer Centre, University Health Network, University of Toronto, Toronto, Ontario, Canada

^12^ Division of Urology, University of Toronto, Toronto, Ontario, Canada

^13^ Division of Urology, Mount Sinai Hospital, Toronto, Ontario, Canada

**Corresponding Author:**

Renil S. Titus

Department of Urology

Houston Methodist Hospital

6560 Fannin Street, Suite 2100

Houston, TX 77030, USA

Tel: (713) 441-6455

Fax: (713) 441-6463

E-mail: [titusrenilsinu@gmail.com](mailto:titusrenilsinu@gmail.com)

**ORCID ID: 0000-0002-5486-749X**

**Twitter: @RenilTitus**

**Supplementary Materials - Index**

| **Supplementary Methods** |  |
| --- | --- |
| Data sources | *pag. 4* |
| Cohort derivation | *pag. 4* |
| Outcomes  Exposure  Covariates | *pag. 4*  *pag. 4*  *pag. 5* |
| Statistical analysis | *pag. 5* |
| **Supplementary Results** |  |
| Baseline demographics and outcomes | *pag. 8* |
| 30-day outcomes  90-day outcomes  1-year outcomes  Subgroup analyses  Sensitivity analyses | *pag. 8*  *pag. 8*  *pag. 9*  *pag. 9*  *pag. 10* |
|  |  |
| **Supplementary Figures and Tables** |  |
| Supplementary Table 1  Supplementary Table 2  Supplementary Table 3  Supplementary Table 4  Supplementary Table 5  Supplementary Table 6  Supplementary Table 7  Supplementary Table 8  Supplementary Table 9  Supplementary Table 10  Supplementary Figure 1  Supplementary Figure 2  **References** | *pag. 11*  *pag. 18*  *pag. 20*  *pag. 21*  *pag. 25*  *pag. 27*  *pag. 28*  *pag. 29*  *pag. 31*  *pag. 33*  *pag. 34*  *pag. 35*  *pag. 36* |
|  |  |
|  |  |
|  |  |
|  |  |
|  |  |
|  |  |
|  |  |
|  |  |
|  |  |

**Supplementary Methods**

*Data sources*

Patient, surgeon, and hospital characteristics were ascertained by linking various healthcare administrative Institute for Clinical Evaluative Sciences (IC/ES) databases: the Canadian Institute for Health Information (CIHI) Discharge Abstract Database (DAD), the OHIP, the CIHI National Ambulatory Care Reporting System (NACRS), the Same Day Surgery (SDS), the Registered Persons Database (RPDB), the Local Health Integration Network (LHIN), the ICES Physician Database (IPDB), and the Facilities (INST) database.

*Cohort derivation*

We identified patients who underwent at least one of the 26 procedures (Supplementary Table-2) during the study interval (n=1,322,525). We excluded patients for whom details of the operating surgeon or anesthesiologist could not be identified (n=95,725), were ≤18-years (n=29,187), underwent multiple procedures performed the same day (n=27,802), without discharge details (n=2,618), with unreliable combinations of specialty and procedure (n=824), non-Ontario-residents (n=340), and whose date of death preceded the surgery (n=318). The overall cohort included 1,165,711 unique patients after excluding 156,814(11.86%). (Supplementary Figure-1)

*Outcomes*

Primary *a priori* outcomes was a composite of 30-day postoperative death, complications, and readmissions, as previously detailed.^1–5^ Postoperative complications (major-morbidity) were defined previously.^6^ All outcomes were ascertained from administrative data using standardized procedural/diagnostic codes.

*Exposure*

Exposure groups were patients undergoing the index procedure in an academic versus non-academic hospital. Based on previous ICES publications,^7,8^ academic hospitals were identified using a list published by Health Force Ontario.^9^ Canada’s Academic Health Science Centers (AHSCs) are composed of health sciences university’s faculty of medicine linked to at least one academic healthcare organizations (Supplementary Table-3).^10^

*Covariates*

Patient-related variables included age, sex, comorbidities (Johns Hopkins Aggregate Diagnosis Group [ADG]), rurality, and socioeconomic status. Surgeon-related variables included age, sex, specialty, annual case volume, and years in practice. Anesthesiologist-related variables included age, sex, annual case volume, and years in practice. Facility- and treatment-related covariates included hospital size (number of beds), admission route (elective vs. urgent), cancer-related procedure (OHIP diagnostic codes 140-239.9), case-complexity, year and surgery duration.

*Statistical analyses*

We descriptively compared patients, physicians, and treatment characteristics by academic status. For the large sample, we compared groups using standardized differences, with significance at 0.10.^11^ Crude event rates were reported for binary outcomes, while means were reported for continuous outcomes. Adjusted event rates and means were derived using multivariable generalized estimating equations (GEE) accounting for patient, surgeon, anesthesiologist, and hospital-level covariates, with clustering on procedure. For binary outcomes, we employed Poisson distribution models with log link. For continuous outcomes, we used negative binomial models with log link. We used models with a logit link to calculate adjusted relative effects; presented as adjusted odds ratio (aOR) for binary outcomes or adjusted relative risk (aRR) for continuous outcomes. We adjusted our estimates by considering the median value of continuous covariates and the third quartile/quintile of categorical covariates.

Subgroup analyses assessing for interaction between patient, physician, and treatment characteristics and the association between academic designation and outcomes was done by examining the effect of hospital size into categories: <250, 250-349, and ≥350 beds and stratified analysis based on surgical urgency (elective vs. emergent), case-complexity (low vs. high), and cancer vs. non-cancer surgery. Further sensitivity analyses by including duration of surgery as a covariate and secondly, excluding physician characteristics from all models were also conducted (Supplementary Tables:6-10, Supplementary Figure-2). All analyses were performed using SAS Enterprise Guide 6.1 (SAS Institute Inc., Cary, NC, USA).

**Supplementary Results**

*Baseline demographics and outcomes*

The final cohort included 1,165,711 patients-392,839 and 772,872 underwent surgery at 29 academic and 126 non-academic hospitals, respectively with a mean age of 59 years( Supplementary Table-1). Most patients were female (61.9%) and lived in urban areas (88.5%). Most surgeries were elective (79.9%) and considered of high complexity (64.9%). More academic surgeons were at the highest quartile for annual case-volume (4^th^ quartile, 32.7%), compared to non-academic surgeons (21.7%). More high-complexity surgeries were undertaken at academic versus non-academic hospitals (75.6% versus 59.4%). A greater proportion of cancer surgeries were at academic versus non-academic hospitals (19.7%v. 11.5%). Mean duration of surgery was longer in academic versus non-academic hospitals (152.6 v. 107.4 minutes).

*30-day outcomes*

At 30-days postoperatively, the crude event rate of the primary outcome was higher for academic versus non-academic hospitals (11.6% v. 10.4%)(Supplementary Table-4). After GEE modeling, the adjusted event rate of the primary outcome remained higher for academic hospitals (10.4% [95%Confidence-Interval (CI): 8.1-13.3]) than for non-academic hospitals (9.3% [95%CI: 6.7-12.9])(Supplementary Table-5). No association was found between academic designation and 30-day mortality (aOR 1.05[95%CI: 0.88-1.26]), nor 30-day complications (aOR 1.09 [95%CI: 0.91-1.31]).

*90-day outcomes*

At 90-days postoperatively, the crude event rate of the primary outcome was higher for academic versus non-academic hospitals (15.5% v 13.6%)(Supplementary Table-4). After GEE modeling, the adjusted event rate of the primary outcome was still higher for academic hospitals (13.6% [95%CI: 11.1-16.7]) than for non-academic hospitals (11.9%[95%CI: 9.3-15.2])(Supplementary Table-5). No association was found between 90-day mortality (aOR 1.13 [95%CI: 0.95-1.36]), nor 90-day complications (aOR 1.10[95%CI: 0.92-1.31]) among patients receiving surgery at academic hospitals.

*1-year outcomes*

At 1-year postoperatively, the crude event rate of the primary outcome was higher for academic hospitals (27.1%) than for non-academic hospitals (23.9%)(Supplementary Table-4). After GEE modeling, the adjusted event rate of the primary outcome was still higher for academic hospitals (24.2% [95%CI: 21.5-27.2]) than for non-academic hospitals (21.4%[95%CI: 19.4-23.5])(Supplementary Table-5). Patients receiving surgery at academic hospitals were not associated with 1-year complications (aOR 1.10[95%CI: 0.94-1.28]).

*Subgroup analyses*

In subgroup analyses utilizing the primary 30-day composite outcome, we found significant heterogeneity by surgeon age, surgeon years in practice, case complexity, and cancer-related surgery (Supplementary Figure-2). In particular, the magnitude of effect, with better outcomes seen among patients treated at community hospitals was larger than patients treated by surgeons aged 51-60 years old (aOR 1.23 [95%CI: 1.10-1.37]; *P* for interaction = 0.004), surgeons >15 years in practice (aOR 1.19 [95%CI: 1.06-1.33]; *P* for interaction = 0.003), those undergoing high complexity procedures (aOR 1.19 [95%CI: 1.12-1.26]; *P* for interaction = 0.042), and those undergoing cancer-related surgery (aOR 1.15 [95%CI: 1.05-1.26]; *P* for interaction < 0.001), demonstrated an increased likelihood of adverse postoperative outcomes for patients receiving surgery at academic hospitals. While there was no significant heterogeneity of effect between hospital type and patient age, patient comorbidity, surgeon specialty, surgeon sex, surgeon volume, anesthesiologist age, surgical procedure type, number of hospital beds, and era of surgery, certain subgroups demonstrated an increased likelihood of adverse postoperative outcomes for patients receiving surgery at academic hospitals.

*Sensitivity analyses*

In the sensitivity analysis accounting for duration of surgery, academic designation and the composite primary outcome were not associated at 30- or 90-day or 1-year (Supplementary Table-7). In the sensitivity analysis excluding physician characteristics, conclusions were concordant with the primary analysis, except for the 90-day composite outcome losing statistical significance (Supplementary Tables-8, 9). Lastly, the sensitivity analysis excluding physician characteristics but including operative time found a decreased likelihood of 30-day adverse postoperative outcomes for patients receiving surgery at academic hospitals (aOR 0.81 [95%CI: 0.72-0.92] *P* = 0.0015) (Supplementary Table-10).

This study has several limitations. First, we did not have data on reasons for readmission. Second, differences in outcomes could represent unmeasured confounding, related to inherent differences in disease, patient and case complexity between academic and non-academic facilities. As previously stated, our differences in long-term outcomes are likely to represent variations in patient and disease characteristics, postoperative care and/or social determinants of health, which we cannot adjust given the limitations of our database. Likewise, we were also unable to capture granular metrics of case complexity. Third, we did not have data on the technical skills of surgeons or the degree of trainee involvement. Fourth, given differences in healthcare systems, our findings might not apply to the U.S. Fifth, we cannot assess for referral bias, by which more difficult cases are referred to academic hospitals. Sixth, we did not have data on postoperative practices that could have affected long-term outcomes, such as post-discharge rehabilitation. Lastly, the procedure types studied in this cohort commonly have increased complications, and therefore our findings may not necessarily apply to other, less common, or predominantly complex cases. Nonetheless, the present study has the strength of including adult patients of all ages, a larger number of index procedures, with a longer follow-up than prior studies.

**Supplementary Figures and Tables**

**Supplementary Table 1:** Baseline Characteristics of Study Cohort, by Hospital Type

| **Variable** | **Label or value** | **Academic (n=392,839)** | **Non-academic (n=772,872)** | **Total (n=1,165,711)** | **P value** | **Standardized Difference** |
| --- | --- | --- | --- | --- | --- | --- |
| **Patient characteristics** | | | | | | |
| Age | Mean (SD), years | 58.7 (16.7) | 59.1 (17.6) | 59.0 (17.3) | <.0001 | 0.021 |
|  | Median (IQR), years | 60 (47-71) | 61 (46-73) | 60 (47-72) | <.0001 | 0.026 |
| Sex, n (%) | Female | 234,560 (59.7%) | 486,655 (63.0%) | 721,215 (61.9%) | <.0001 | 0.067 |
|  | Male | 158,279 (40.3%) | 286,217 (37.0%) | 444,496 (38.1%) |  | 0.067 |
| Comorbidity, n (%) | ADG 0-5 | 100,845 (25.7%) | 203,995 (26.4%) | 304,840 (26.2%) | <.0001 | 0.016 |
|  | ADG 6-7 | 93,130 (23.7%) | 185,127 (24.0%) | 278,257 (23.9%) |  | 0.006 |
|  | ADG 8-10 | 117,752 (30.0%) | 232,562 (30.1%) | 350,314 (30.1%) |  | 0.003 |
|  | AGD>=11 | 81,112 (20.6%) | 151,188 (19.6%) | 232,300 (19.9%) |  | 0.027 |
| Rurality, n (%) | Urban | 341,644 (87.0%) | 689,431 (89.2%) | 1,031,075 (88.5%) | <.0001 | 0.069 |
|  | Rural | 51,195 (13.0%) | 83,441 (10.8%) | 134,636 (11.5%) |  | 0.069 |
| Income quintile, n (%) | 1 - Lowest | 75,765 (19.3%) | 146,546 (19.0%) | 222,311 (19.1%) | <.0001 | 0.008 |
|  | 2 - | 79,206 (20.2%) | 156,317 (20.2%) | 235,523 (20.2%) |  | 0.002 |
|  | 3 - | 76,018 (19.4%) | 158,154 (20.5%) | 234,172 (20.1%) |  | 0.028 |
|  | 4 - | 76,940 (19.6%) | 160,797 (20.8%) | 237,737 (20.4%) |  | 0.030 |
|  | 5 - Highest | 84,910 (21.6%) | 151,058 (19.5%) | 235,968 (20.2%) |  | 0.051 |
| **Surgeon characteristics** | | | | | | |
| Age | Mean (SD), years | 49.3 (9.8) | 49.1 (9.3) | 49.2 (9.5) | <.0001 | 0.022 |
|  | Median (IQR), years | 48 (41-57) | 48 (42-56) | 48 (41-56) | <.0001 | 0.010 |
| Sex, n (%) | Female | 56,591 (14.4%) | 94,463 (12.2%) | 151,054 (13.0%) | <.0001 | 0.064 |
|  | Male | 336,248 (85.6%) | 678,409 (87.8%) | 1,014,657 (87.0%) |  | 0.064 |
| Annual case volume (quartiles), n (%) | 1 - Lowest | 92,498 (23.5%) | 189,437 (24.5%) | 281,935 (24.2%) | <.0001 | 0.023 |
|  | 2- | 87,099 (22.2%) | 215,203 (27.8%) | 302,302 (25.9%) |  | 0.131 |
|  | 3- | 84,929 (21.6%) | 200,374 (25.9%) | 285,303 (24.5%) |  | 0.101 |
|  | 4 - Highest | 128,313 (32.7%) | 167,858 (21.7%) | 296,171 (25.4%) |  | 0.248 |
| Years in practice | Mean (SD), years | 16.1 (8.8) | 15.5 (8.5) | 15.7 (8.6) | <.0001 | 0.066 |
|  | Median (IQR), years | 17 (8-23) | 16 (8-22) | 17 (8-23) | <.0001 | 0.062 |
| Specialty, n (%) | Cardiothoracic Surgery | 1,236 (0.3%) | 2,742 (0.4%) | 3,978 (0.3%) | <.0001 | 0.007 |
|  | General Surgery | 105,796 (26.9%) | 280,025 (36.2%) | 385,821 (33.1%) |  | 0.201 |
|  | Neurosurgery | 45,495 (11.6%) | 13,417 (1.7%) | 58,912 (5.1%) |  | 0.403 |
|  | Obstetrics and Gynecology | 48,197 (12.3%) | 93,172 (12.1%) | 141,369 (12.1%) |  | 0.007 |
|  | Orthopedic Surgery | 134,530 (34.2%) | 257,420 (33.3%) | 391,950 (33.6%) |  | 0.020 |
|  | Otolaryngology | 8,632 (2.2%) | 10,486 (1.4%) | 19,118 (1.6%) |  | 0.064 |
|  | Plastic Surgery | 14,202 (3.6%) | 40,826 (5.3%) | 55,028 (4.7%) |  | 0.081 |
|  | Thoracic Surgery | 7,843 (2.0%) | 7,192 (0.9%) | 15,035 (1.3%) |  | 0.089 |
|  | Urology | 24,783 (6.3%) | 65,636 (8.5%) | 90,419 (7.8%) |  | 0.083 |
|  | Vascular Surgery | 2,125 (0.5%) | 1,956 (0.3%) | 4,081 (0.4%) |  | 0.046 |
| **Anesthesiologist characteristics** | | | | | | |
| Age | Mean (SD), years | 47.8 (9.9) | 49.5 (10.2) | 48.9 (10.1) | <.0001 | 0.168 |
|  | Median (IQR), years | 47 (40-55) | 49 (41-57) | 48 (41-57) | <.0001 | 0.163 |
| Sex, n (%) | Female | 113,746 (29.0%) | 198,076 (25.6%) | 311,822 (26.7%) | <.0001 | 0.075 |
|  | Male | 279,093 (71.0%) | 574,796 (74.4%) | 853,889 (73.3%) |  | 0.075 |
| Annual case volume (quartiles), n (%) | 1 - Lowest | 133,001 (33.9%) | 138,563 (17.9%) | 271,564 (23.3%) | <.0001 | 0.370 |
|  | 2 - | 118,373 (30.1%) | 187,639 (24.3%) | 306,012 (26.3%) |  | 0.132 |
|  | 3 - | 85,572 (21.8%) | 210,633 (27.3%) | 296,205 (25.4%) |  | 0.127 |
|  | 4 - Highest | 55,893 (14.2%) | 236,037 (30.5%) | 291,930 (25.0%) |  | 0.399 |
| Years in practice | Mean (SD), years | 14.2 (9.3) | 14.8 (9.4) | 14.6 (9.4) | <.0001 | 0.058 |
|  | Median (IQR), years | 13 (6-22) | 14 (6-23) | 14 (6-22) | <.0001 | 0.056 |
| **Treatment characteristics** | | | | | | |
| Total hospital beds | Missing | 81,493 (20.7%) | 177,987 (23.0%) | 259,480 (22.3%) | <.0001 | 0.055 |
|  | <249 | 28,081 (7.1%) | 341,231 (44.2%) | 369,312 (31.7%) |  | 0.935 |
|  | 250-349 | 69,000 (17.6%) | 177,480 (23.0%) | 246,480 (21.1%) |  | 0.135 |
|  | ≥350 | 214,265 (54.5%) | 76,174 (9.9%) | 290,439 (24.9%) |  | 1.089 |
| Surgical procedure type, n (%) | Elective | 312,034 (79.4%) | 619,285 (80.1%) | 931,319 (79.9%) | <.0001 | 0.017 |
|  | Urgent | 80,805 (20.6%) | 153,587 (19.9%) | 234,392 (20.1%) |  | 0.017 |
| Cancer-related procedure^a^, n (%) | Yes | 77,287 (19.7%) | 88,680 (11.5%) | 165,967 (14.2%) | <.0001 | 0.228 |
|  | No | 315,552 (80.3%) | 684,192 (88.5%) | 999,744 (85.8%) |  | 0.228 |
| Case complexity, n (%) | Low | 95,747 (24.4%) | 313,835 (40.6%) | 409,582 (35.1%) | <.0001 | 0.352 |
|  | High | 297,092 (75.6%) | 459,037 (59.4%) | 756,129 (64.9%) |  | 0.352 |
| Duration of index surgery | Missing on duration, n (%) | 12,254 (3.1%) | 53,264 (6.9%) | 65,518 (5.6%) | <.0001 | 0.174 |
|  | Non-missing on duration, n (%) | 380,585 (96.9%) | 719,608 (93.1%) | 1,100,193 (94.4%) |  | 0.174 |
|  | Mean (SD), minutes | 152.6 (110.9) | 107.4 (96.9) | 123.1 (104.2) | <.0001 | 0.434 |
|  | Median (IQR), minutes | 127 (93-185) | 95 (68-130) | 105 (75-148) | <.0001 | 0.623 |
| Year of index surgery, n (%) | 2007 | 34,587 (8.8%) | 65,271 (8.4%) | 99,858 (8.6%) | <.0001 | 0.013 |
|  | 2008 | 33,629 (8.6%) | 63,344 (8.2%) | 96,973 (8.3%) |  | 0.013 |
|  | 2009 | 33,539 (8.5%) | 63,275 (8.2%) | 96,814 (8.3%) |  | 0.013 |
|  | 2010 | 33,446 (8.5%) | 62,385 (8.1%) | 95,831 (8.2%) |  | 0.016 |
|  | 2011 | 33,485 (8.5%) | 63,126 (8.2%) | 96,611 (8.3%) |  | 0.013 |
|  | 2012 | 32,322 (8.2%) | 61,847 (8.0%) | 94,169 (8.1%) |  | 0.008 |
|  | 2013 | 33,476 (8.5%) | 63,866 (8.3%) | 97,342 (8.4%) |  | 0.009 |
|  | 2014 | 32,596 (8.3%) | 62,986 (8.1%) | 95,582 (8.2%) |  | 0.005 |
|  | 2015 | 31,281 (8.0%) | 60,788 (7.9%) | 92,069 (7.9%) |  | 0.004 |
|  | 2016 | 27,966 (7.1%) | 57,933 (7.5%) | 85,899 (7.4%) |  | 0.014 |
|  | 2017 | 24,128 (6.1%) | 53,499 (6.9%) | 77,627 (6.7%) |  | 0.032 |
|  | 2018 | 22,326 (5.7%) | 49,392 (6.4%) | 71,718 (6.2%) |  | 0.030 |
|  | 2019 | 20,058 (5.1%) | 45,160 (5.8%) | 65,218 (5.6%) |  | 0.032 |

SD: standard deviation, IQR: interquartile range

^a^ as per OHIP diagnostic codes 140-239.9

**Supplementary Table 2. Included procedures and corresponding OHIP fee codes**

|  | **Surgery** | **OHIP Fee Code** |
| --- | --- | --- |
| A. | Coronary artery bypass grafting | R742, R743 |
| B. | Femoral-popliteal bypass | R791, R794 |
| C. | Abdominal aortic aneurysm repair | R802, R817, R877 |
| D. | Appendectomy | S205 |
| E. | Cholecystectomy | S287 |
| F. | Gastric bypass Roux-en-Y | S120 |
| G. | Colon resection | S166, S167, S168, S169, S170, S171, S173, S174, S213, S214, S215, S216, S217 |
| H. | Liver resection | S267, S269, S270, S271, S275 |
| I. | Hysterectomy | S757, S816, S758, S759, S710, S763 |
| J. | Spinal surgery |  |
| i. | Anterior decompression | N500, N501, N502, N503, N504, N505, N506, N507, N508, N579 |
| ii. | Anterior arthrodesis | N516, N517, N518, N559, N580 |
| iii. | Posterior decompression | N509, N510, N520, N511, N512, N524 |
| iv. | Posterior arthrodesis | N515, N519, N514, N581, N582,  N533 |
| K. | Craniotomy for brain tumor/biopsy | N102, N103, N151, N152, N153 / N113 |
| L. | Knee replacement TKR | R441 |
| M. | Hip replacement THR | R440 |
| N. | Open repair femoral neck/femoral shaft fracture | F099, F100, F101/F096 |
| O. | Total thyroidectomy | S788 |
| P. | Neck dissection | R910, R915 |
| Q. | Lung resection pneumonectomy, lobectomy or segmental resection | M142, M143, M144 |
| R. | Radical cystectomy | S440, S453 |
| S. | Radical prostatectomy | S651 |
| T. | Transurethral resection of prostate | S655 |
| U. | Carpal tunnel release | N290 |
| V. | Breast reduction | R110 |

**Supplementary Table 3:** Academic Hospitals in Ontario^25^

| **Hamilton** | **Hamilton Health Sciences Corporation** |
| --- | --- |
|  | - Hamilton General Hospital |
|  | - Juravinski Hospital and Cancer Centre |
|  | - McMaster University Medical Centre |
|  | - St. Peter's Hospital |
|  | - West Lincoln Memorial Hospital (Grimsby) |
| **Hamilton** | **St. Joseph’s Healthcare Hamilton** |
|  | - Charlton Campus |
| **Kingston** | **Kingston Health Sciences Centre** |
|  | **-**Hotel Dieu Hospital |
|  | - Kingston General Hospital |
| **London** | **London Health Sciences Centre** |
|  | - University Hospital |
|  | - Victoria Hospital |
| **London** | **St. Joseph's Health Care, London** |
|  | - St. Joseph's Hospital |
| **Ottawa** | **Children's Hospital of Eastern Ontario-Ottawa Children's Treatment Centre** |
| **Ottawa** | **Hôpital Montfort** |
| **Ottawa** | **The Ottawa Hospital / L'Hôpital D'Ottawa** |
|  | - Civic Campus |
|  | - General Campus |
| **Sudbury** | **Health Sciences North** |
|  | - Ramsey Lake Health Centre |
|  | - Kirkwood site |
| **Thunder Bay** | **Thunder Bay Regional Health Sciences Centre** |
| **Toronto** | **Sinai Health System** |
|  | - Mount Sinai Hospital |
| **Toronto** | **University Health Network** |
|  | - Toronto General Hospital |
|  | - Toronto Western Hospital |
|  | - Princess Margaret Cancer Centre |
| **Toronto** | **Sunnybrook Health Sciences Centre** |
|  | - Bayview Campus |
|  | - Holland Centre |
| **Toronto** | **Hospital for Sick Children (The)** |
| **Toronto** | **Women's College Hospital** |
| **Toronto** | **Unity Health Toronto** |
|  | - St. Michael’s Hospital |

**Supplementary Table-4:** Crude Event Rate or Mean of Outcomes within 30- and 90-Days and 1-Year of Index Surgery, by Hospital Type

| **Outcome** | **Label** | **Outcome Time Period** | **Academic Hospital** | **Non-Academic Hospital** | **Overall** | **P value** |
| --- | --- | --- | --- | --- | --- | --- |
| Composite Endpoint | No. (%) | Within 30 Days | 45,508 (11.6%) | 80,554 (10.4%) | 126,062 (10.8%) | <.0001 |
| Death | No. (%) |  | 4,505 (1.15%) | 8,542 (1.11%) | 13,047 (1.12%) | 0.0438 |
| Re-admission | No. (%) |  | 21,654 (5.5%) | 33,475 (4.3%) | 55,129 (4.7%) | <.0001 |
| Complications | No. (%) |  | 25,248 (6.4%) | 48,277 (6.2%) | 73,525 (6.3%) | 0.0001 |
| Hospital Stay (days) | Mean (SD) |  | 4.9 (6.4) | 3.9 (5.3) | 4.3 (5.7) | <.0001 |
| Duration of Index Surgery (minutes) | Mean (SD) |  | 152.6 (110.9) | 107.4 (96.9) | 123.1 (104.2) | <.0001 |
| Composite Endpoint | Rate (%) | Within 90 Days | 60,932 (15.5%) | 104,973 (13.6%) | 165,905 (14.2%) | <.0001 |
| Death | Rate (%) |  | 8,848 (2.3%) | 14,895 (1.9%) | 23,743 (2.0%) | <.0001 |
| Re-admission | Rate (%) |  | 36,995 (9.4%) | 57,258 (7.4%) | 94,253 (8.1%) | <.0001 |
| Complications | Rate (%) |  | 26,639 (6.8%) | 50,371 (6.5%) | 77,010 (6.6%) | <.0001 |
| Hospital Stay (days) | Mean (SD) |  | 6.3 (11.9) | 4.7 (8.9) | 5.2 (10.0) | <.0001 |
| Composite Endpoint | Rate (%) | Within 1 Year | 106,481 (27.1%) | 184,658 (23.9%) | 291,139 (25.0%) | <.0001 |
| Death | Rate (%) |  | 20,140 (5.1%) | 29,849 (3.9%) | 49,989 (4.3%) | <.0001 |
| Re-admission | Rate (%) |  | 83,217 (21.2%) | 137,158 (17.7%) | 220,375 (18.9%) | <.0001 |
| Complications | Rate (%) |  | 32,172 (8.2%) | 60,137 (7.8%) | 92,309 (7.9%) | <.0001 |
| Hospital Stay (days) | Mean (SD) |  | 8.6 (20.5) | 6.3 (14.9) | 7.1 (17.0) | <.0001 |

**Supplementary Table-5:** Adjusted Event Rate or Mean of Outcomes within 30- and 90-Days and 1-Year of Index Surgery, by Hospital Type

| **Outcome** | **Label** | **Outcome Time Period** | **Academic Hospital Adjusted Rate or Mean (95% CI)** | **Non-Academic Hospital Adjusted Rate or Mean (95% CI)** |
| --- | --- | --- | --- | --- |
| Composite Endpoint | Rate (%) | Within 30 Days | 10.4 (8.1-13.3) | 9.3 (6.7-12.9) |
| Death | Rate (%) |  | 0.3 (0.2-0.6) | 0.3 (0.1-0.6) |
| Re-admission | Rate (%) |  | 5.1 (4.3-6.1) | 4.1 (3.5-4.7) |
| Complications | Rate (%) |  | 5.7 (3.9-8.4) | 5.5 (3.2-9.3) |
| Hospital Stay (days) | Mean |  | 4.0 (3.1-5.2) | 3.1 (2.3-4.0) |
| Duration of Surgery (minutes) | Mean |  | 138.5 (115.8-165.7) | 99.3 (82.7-119.3) |
| Composite Endpoint | Rate (%) | Within 90 Days | 13.6 (11.1-16.7) | 11.9 (9.3-15.2) |
| Death | Rate (%) |  | 0.8 (0.4-1.5) | 0.6 (0.3-1.2) |
| Re-admission | Rate (%) |  | 8.2 (6.9-9.8) | 6.6 (5.7-7.7) |
| Complications | Rate (%) |  | 6.0 (4.2-8.7) | 5.7 (3.4-9.5) |
| Hospital Stay (days) | Mean |  | 4.8 (3.6-6.4) | 3.4 (2.7-4.5) |
| Composite Endpoint | Rate (%) | Within 1 Year | 24.2 (21.5-27.2) | 21.4 (19.4-23.5) |
| Death | Rate (%) |  | 2.2 (1.1-4.5) | 1.6 (0.9-2.9) |
| Re-admission | Rate (%) |  | 18.9 (16.0-22.4) | 16.1 (13.2-19.7) |
| Complications | Rate (%) |  | 7.2 (5.3-9.8) | 6.8 (4.5-10.4) |
| Hospital Stay (days) | Mean |  | 6.4 (4.8-8.6) | 4.5 (3.5-5.8) |
| Note: using GEE modeling with clustering on procedure fee code (Poisson distribution with log link for binary outcomes and Negative Binominal with log link for continuous outcomes), adjusted for surgeon age (median age), surgeon annual case volume (3rd quartile), surgeon years of practice (median value), anesthesiologist age (median age), anesthesiologist annual case volume (3rd quartile), anesthesiologist years of practice (median value), patient age (median age), patient comorbidity (ADG=8-10), rurality (urban), income quintile (3rd quintile) , and hospital status (academic). | | | | |

**Supplementary Table 6:** Definitions of low- and high-complexity surgical procedures for subgroup analyses

| **Category** | **Low complexity procedures** | **High complexity procedures** |
| --- | --- | --- |
| Definition | Procedures with little variability from case to case (“straightforward” procedures) and those which are performed by a significant proportion of surgeons within that specialty | Remaining procedures not categorized as low complexity |
| Included procedures: | Appendectomy, cholecystectomy, carpal tunnel release | Coronary artery bypass grafting, femoral-popliteal bypass, abdominal aortic aneurysm repair, gastric bypass, colon resection, liver resection, anterior or posterior spinal decompression, anterior or posterior spinal arthrodesis, craniotomy for brain tumor, total knee replacement, total hip replacement, open repair of femoral neck or shaft fracture, total thyroidectomy, neck dissection, lung resection, radical cystectomy |

**Supplementary Table 7:** Sensitivity Analysis #1: Multivariable Generalized Estimating Equation Regression Models, with Clustering based on Procedure Fee Code for the Composite Outcome within 30- and 90-Days and 1-Year of Index Surgery, by Hospital Type

|  | **Outcome within 30 Days** | | **Outcome within 90 Days** | | **Outcome within 1 Year** | |
| --- | --- | --- | --- | --- | --- | --- |
| **Outcome** | **Academic vs. Non-Academic Hospital aOR (95% CI)** | **P value** | **Academic vs. Non-Academic Hospital aOR (95% CI)** | **P value** | **Academic vs. Non-Academic Hospital aOR (95% CI)** | **P value** |
| Composite Endpoint | 0.97 (0.88-1.07) | 0.5881 | 0.99 (0.90-1.10) | 0.9157 | 1.01 (0.94-1.09) | 0.7363 |
| * Adjusted odds ratio (aOR) for binary outcomes. | | | | | | |
| Note: using GEE modeling dealing with clustering based on procedure fee code (logistic regression with binomial distribution with logit link for binary outcomes), adjusted for surgeon age (continuous), surgeon sex, surgeon annual case volume (quartiles), surgeon specialty, surgeon years of practice (continuous), anesthesiologist age (continuous), anesthesiologist sex, anesthesiologist annual case volume (quartiles), anesthesiologist years of practice (continuous), patient age (continuous), patient sex, patient comorbidity (categorical), rurality (rural vs. urban), income quintile (quintiles) , LHIN, hospital status (academic vs. community), and index year, **as well as duration of index surgery.** | | | | | | |

**Supplementary Table 8:** Sensitivity analysis #2 - Adjusted Event Rate or Mean of Outcomes within 30- and 90-Days and 1-Year of Index Surgery, by Hospital Type *(excluding surgeon and anesthesiologist characteristics)*

| **Outcome** | **Label** | **Outcome Time Period** | **Academic Hospital Adjusted Rate or Mean (95% CI)** | **Non-Academic Hospital Adjusted Rate or Mean (95% CI)** |
| --- | --- | --- | --- | --- |
| Composite Endpoint | Rate (%) | Within 30 Days | 10.9 (8.5-14.1) | 10.0 (7.5-13.4) |
| Death | Rate (%) |  | 0.4 (0.2-0.8) | 0.3 (0.2-0.7) |
| Re-admission | Rate (%) |  | 5.3 (4.3-6.5) | 4.2 (3.7-4.9) |
| Complications | Rate (%) |  | 6.0 (4.4-8.3) | 6.0 (3.9-9.1) |
| Hospital Stay (days) | Mean |  | 4.8 (3.9-5.9) | 3.6 (2.8-4.5) |
| Duration of Surgery (minutes) | Mean |  | 157.9 (134.8-185.0) | 106.8 (91.3-125.0) |
| Composite Endpoint | Rate (%) | Within 90 Days | 14.7 (11.8-18.5) | 12.9 (10.3-16.2) |
| Death | Rate (%) |  | 0.9 (0.4-1.8) | 0.6 (0.3-1.2) |
| Re-admission | Rate (%) |  | 9.2 (7.6-11.1) | 7.2 (6.2-8.2) |
| Complications | Rate (%) |  | 6.4 (4.7-8.7) | 6.2 (4.2-9.3) |
| Hospital Stay (days) | Mean |  | 6.1 (4.7-7.9) | 4.2 (3.3-5.4) |
| Composite Endpoint | Rate (%) | Within 1 Year | 26.3 (23.0-30.1) | 23.1 (20.8-25.8) |
| Death | Rate (%) |  | 2.7 (1.3-5.6) | 1.7 (1.0-3.0) |
| Re-admission | Rate (%) |  | 21.0 (18.4-24.0) | 17.5 (14.8-20.6) |
| Complications | Rate (%) |  | 7.7 (5.8-10.2) | 7.5 (5.3-10.5) |
| Hospital Stay (days) | Mean |  | 8.3 (6.4-10.9) | 5.6 (4.4-7.2) |
| Note: using GEE modeling dealing with clustering based on procedure fee code (Poisson distribution with log link for binary outcomes and Negative Binominal with log link for continuous outcomes), adjusted for patient age (using the median age), patient comorbidity (using ADG=8-10), rurality (using urban), income quintile (using 3rd quintile), and hospital status (using academic). | | | | |

**Supplementary Table 9:** Sensitivity analysis #2 - Multivariable Generalized Estimating Equation Regression Models, with Clustering based on Procedure Fee Code for Outcomes within 30- and 90-Days and 1-Year of Index Surgery, by Hospital Type *(excluding surgeon and anesthesiologist characteristics)*

|  |  | **Outcome within 30 Days** | | **Outcome within 90 Days** | | **Outcome within 1 Year** | |
| --- | --- | --- | --- | --- | --- | --- | --- |
| **Model No.** | **Outcome** | **Academic vs. Non-academic Hospital aOR/aRR (95% CI)** | **P value** | **Academic vs. Non-Academic Hospital aOR/aRR (95% CI)** | **P value** | **Academic vs. Non-Academic Hospital aOR/aRR (95% CI)** | **P value** |
| 1 | Composite Endpoint | 1.10 (0.95-1.29) | 0.2008 | 1.17 (1.00-1.37) | 0.0506 | 1.19 (1.04-1.37) | 0.0137 |
| 2 | Death | 1.18 (0.87-1.60) | 0.2882 | 1.39 (0.92-2.10) | 0.1142 | 1.65 (1.01-2.70) | 0.0475 |
| 3 | Re-admission | 1.27 (1.12-1.43) | 0.0002 | 1.32 (1.12-1.54) | 0.0007 | 1.26 (1.09-1.47) | 0.0025 |
| 4 | Complications | 1.01 (0.81-1.25) | 0.9429 | 1.02 (0.83-1.26) | 0.8391 | 1.03 (0.86-1.23) | 0.7316 |
| 5 | Hospital Stay (days) | 1.33 (1.15-1.54) | 0.0001 | 1.45 (1.22-1.72) | <0.0001 | 1.48 (1.24-1.78) | <0.0001 |
| 6 | Duration of Surgery | 1.48 (1.28-1.71) | <0.0001 | N/A | N/A | N/A | N/A |
| * Adjusted odds ratio (aOR) for binary outcomes and adjusted relative risk (aRR) for continuous outcomes. | | | | | |  |  |
| Note: using GEE modeling dealing with clustering based on procedure fee code (logistic regression with binomial distribution with logit link for binary outcomes, and negative binomial distribution with log link for continuous outcomes), adjusted for patient age (continuous), patient sex, patient comorbidity (categorical), rurality (rural vs. urban), income quintile (quintiles), LHIN, hospital status (academic vs. non-academic), and index year. | | | | | | | |

**Supplementary Table 10:** Sensitivity Analysis #2 - Multivariable Generalized Estimating Equation Regression Models, with Clustering based on Procedure Fee Code for the Composite Outcome within 30- and 90-Days and 1-Year of Index Surgery, by Hospital Type *(excluding surgeon and anesthesiologist characteristics)*

|  | **Outcome within 30 Days** | | **Outcome within 90 Days** | | **Outcome within 1 Year** | |
| --- | --- | --- | --- | --- | --- | --- |
| **Outcome** | **Academic vs. Non-Academic Hospital aOR (95% CI)** | **P value** | **Academic vs. Non-Academic Hospital aOR (95% CI)** | **P value** | **Academic vs. Non-Academic Hospital aOR (95% CI)** | **P value** |
| Composite Endpoint | 0.81 (0.72-0.92) | 0.0015 | 0.88 (0.77-1.01) | 0.0797 | 0.98 (0.87-1.10) | 0.7358 |
| * Adjusted odds ratio (aOR) for binary outcomes. | | | | | | |
| Note: using GEE modeling dealing with clustering based on procedure fee code (logistic regression with binomial distribution with logit link for binary outcomes), adjusted for patient age (continuous), patient sex, patient comorbidity (categorical), rurality (rural vs. urban), income quintile (quintiles), LHIN, hospital status (academic vs. community), and index year, **as well as duration of index surgery.** | | | | | | |

**Supplementary Figure 1:** Cohort selection

Patients whose surgeon did not have valid age and/or sex

n = 48,243

Patients whose anesthesiologist did not have valid age and/or sex

n = 47,482

Patients <18 years old

n = 29,187

Patients with multiple surgical procedures on index date

n = 27,802

Patients with missing linked data

n = 2,618

Patients not residents of Ontario

n = 340

Patients with date of death preceding date of index surgery

n = 318

Patients with invalid combinations of procedure and specialty or sex

n = 824

Patients who had at least one of the 26 indexed surgical procedures

n = 1,322,525

Final cohort

n = 1,165,711

Academic

n = 392,839

Non-Academic

n = 772,872

**Supplementary Figure 2:** Forest plot of subgroup analysis assessing the association between academic designation, stratified patient, surgeon, anesthesiologist, and treatment characteristics.

‡, Results for the analysis were from an ordinary logistic regression, because the GEE modeling did not converge

**REFERENCES:**

1. Jerath A, Satkunasivam R, Kaneshwaran K, et al. Association Between Anesthesiologist Sex and Patients’ Postoperative Outcomes: A Population-based Cohort Study. *Ann Surg*. 2024;279(4):569-574. doi:10.1097/SLA.0000000000006217

2. Wallis CJ, Ravi B, Coburn N, Nam RK, Detsky AS, Satkunasivam R. Comparison of postoperative outcomes among patients treated by male and female surgeons: a population based matched cohort study. *BMJ*. 2017;359:j4366. doi:10.1136/bmj.j4366

3. Wallis CJD, Jerath A, Coburn N, et al. Association of Surgeon-Patient Sex Concordance With Postoperative Outcomes. *JAMA Surg*. 2022;157(2):146-156. doi:10.1001/jamasurg.2021.6339

4. Wallis CJ, Jerath A, Ikesu R, et al. Association between patient-surgeon gender concordance and mortality after surgery in the United States: retrospective observational study. *BMJ*. 2023;383:e075484. doi:10.1136/bmj-2023-075484

5. Satkunasivam R, Klaassen Z, Ravi B, et al. Relation between surgeon age and postoperative outcomes: a population-based cohort study. *CMAJ Can Med Assoc J J Assoc Medicale Can*. 2020;192(15):E385-E392. doi:10.1503/cmaj.190820

6. Outcomes of Daytime Procedures Performed by Attending Surgeons after Night Work | New England Journal of Medicine. Accessed April 19, 2025. https://www.nejm.org/doi/full/10.1056/NEJMsa1415994

7. McGee J, Winick-Ng J, McClure JA, et al. Resident Trainees Increase Surgical Time: A Comparison of Obstetric and Gynaecologic Procedures in Academic Versus Community Hospitals. *J Obstet Gynaecol Can JOGC J Obstet Gynecol Can JOGC*. 2020;42(4):430-438.e2. doi:10.1016/j.jogc.2019.08.042

8. Vinden C, Malthaner R, McGee J, et al. Teaching surgery takes time: the impact of surgical education on time in the operating room. *Can J Surg J Can Chir*. 2016;59(2):87-92. doi:10.1503/cjs.017515

9. Resources. Accessed October 22, 2024. https://www.healthforceontario.ca/en/Home/All_Programs/Clerkship_Travel_Program/Resources

10. Brimacombe GG. *Three Missions, One Future-: Optimizing the Performance of Canada’s Academic Health Sciences Centres*. Association of Canadian Academic Healthcare Organizations; 2010.

11. Austin PC. Using the Standardized Difference to Compare the Prevalence of a Binary Variable Between Two Groups in Observational Research. *Commun Stat - Simul Comput*. Published online May 14, 2009. doi:10.1080/03610910902859574
